# Diverse sampling programs highlight pulses of *Velella velella* along the US West Coast

**DOI:** 10.1093/plankt/fbag044

**Published:** 2026-06-13

**Authors:** Megan A Cimino, John A Conroy, Ryan Gasbarro, Michael G Jacox, Brian Hoover, Jarrod A Santora, Danial G Palance, Emily C Nazario, Isaac Schroeder, Adena Schonfeld, Allison Cluett, Mercedes Pozo Buil, Nerea Lezama-Ochoa, Elliott L Hazen, Eric Bjorkstedt, Jaime Jahncke, Timothy Jones, Mark D Ohman, Julia Parrish

**Affiliations:** Institute of Marine Sciences, University of California Santa Cruz, 1156 High St., Santa Cruz, CA 95064, USA; Ocean Sciences Department, University of California Santa Cruz, 1156 High St., Santa Cruz, CA 95064, USA; Ocean Sciences Department, University of California Santa Cruz, 1156 High St., Santa Cruz, CA 95064, USA; Institute of Marine Sciences, University of California Santa Cruz, 1156 High St., Santa Cruz, CA 95064, USA; NOAA Southwest Fisheries Science Center, Climate & Ecosystem Group, 99 Pacific St., Monterey, CA 93940, USA; NOAA Physical Sciences Laboratory, Earth System Research Laboratories, 325 Broadway, Boulder, CO 80305, USA; Farallon Institute, 101 H St, Petaluma, CA 94952, USA; NOAA Southwest Fisheries Science Center, Fisheries Ecology Division, 110 McAllister Way, Santa Cruz, CA 95060, USA; Department of Ecology & Evolutionary Biology, University of California Santa Cruz, 130 McAllister Way, Santa Cruz, CA 95060, USA; Department of Ecology & Evolutionary Biology, University of California Santa Cruz, 130 McAllister Way, Santa Cruz, CA 95060, USA; Fisheries Collaborative Program, University of California Santa Cruz, 1156 High St, Santa Cruz, CA 94064, USA; Fisheries Collaborative Program, University of California Santa Cruz, 1156 High St, Santa Cruz, CA 94064, USA; Fisheries Collaborative Program, University of California Santa Cruz, 1156 High St, Santa Cruz, CA 94064, USA; Institute of Marine Sciences, University of California Santa Cruz, 1156 High St., Santa Cruz, CA 95064, USA; Fisheries Collaborative Program, University of California Santa Cruz, 1156 High St, Santa Cruz, CA 94064, USA; NOAA Southwest Fisheries Science Center, Climate & Ecosystem Group, 99 Pacific St., Monterey, CA 93940, USA; Department of Fisheries Biology, California State Polytechnic University, Humboldt, 1 Harpst St, Arcata, CA 95521, USA; Point Blue Conservation Science, 3820 Cypress Drive, Suite #11, Petaluma, CA 94954, USA; British Antarctic Survey, High Cross, Madingley Road, Cambridge CB3 0ET, UK; Scripps Institution of Oceanography, University of California San Diego, 9300 Gilman Drive, La Jolla, CA 92093, USA; College of the Environment, University of Washington, 1492 NE Boat St, Seattle, WA 98105, USA

**Keywords:** pleuston, distribution anomaly, by-the-wind sailor

## Abstract

Sporadic mass strandings of the hydrozoan, *Velella velella*, along the US West Coast fascinate beachcombers and perplex oceanographers. *Velella* often arrive to the coast in the spring concurrent with a shift in onshore winds and after winters with warmer water temperatures. Understanding the factors that bring *Velella* to coastal waters provides new information about the ecological impacts of local and basin-scale environmental variability in the northeast Pacific. We summarize several compelling, non-exclusive hypotheses that could explain the recent increase in *Velella*, including increased population size, larger surface patches and enhanced coastward transport. We compiled a comprehensive dataset spanning planktonic larval to colonial adult life stages from 10 sources covering ~1900–2025. We highlight pulses of *Velella* and an unprecedented number of strandings and at-sea observations since 2014. We found that wind patterns in the central-east Pacific favor transport toward the coast during years of *Velella* presence. However, we found no clear relationship between surface frontal features and *Velella* abundance, nor a consistent association with El Niño. We also identified phalarope abundance as a potential seabird proxy for increased *Velella* abundance at-sea. Finally, we provide recommendations for future monitoring efforts to improve long-term assessments, given the current inability to clearly distinguish among hypotheses.

## INTRODUCTION

“The distribution of the pleuston presents a challenging and difficult problem, containing many whirlpools of error into which incautious investigators may be sucked” (Mackie, 1962).

Episodically present species are valuable indicators of environmental shifts, but long-term data are often sparse, limiting our understanding of their historical fluctuations. In the Northeast Pacific, despite frequent mass strandings of “by-the-wind sailors”, *Velella velella* (hereafter *Velella*), much remains unknown about their life cycle, habitat associations and population dynamics. Given that their distributions have been related to oceanographic and atmospheric conditions (Pires *et al.,* 2018; Jones *et al.,* 2021; Epinoux *et al.,* 2026), *Velella* may provide useful insights into ecosystem variability.

*Velella* is a hydrozoan that alternates between generations of medusae (initially <1 mm) that reside in the subsurface and polyps that form floating colonies on the surface (up to 140 mm) (Bieri, 1977). Relatively little is known about the medusa stage, and contrasting hypotheses suggest medusae may reside in the epipelagic zone (Larson, 1980) or sink to 600–1000 m for reproduction (Woltereck, 1904). Medusae release larvae that ascend to the surface where colonies develop and grow in the pleuston, i.e. at the air–sea interface. Seasonal observations in the Northeast Pacific suggest two generations per year, with surface arrivals concentrated in January and July (Bieri, 1977).

*Velella* are numerous in a convergence zone within the North Pacific Subtropical Gyre between 20 and 40°N (Bieri, 1977; Chong *et al.,* 2023), but understanding of *Velella* distribution across the Northeast Pacific remains incomplete. Colonies with a “left-handed” rigid sail drift left of the downwind direction and dominate observations along the west coast of North America (Bieri, 1959; Mackie, 1962). Here, coastal strandings spanning >400 km (40–49°N) typically coincided with shifts to onshore winds (northwesterly) in April and were preceded by warmer winter sea surface temperature (Jones *et al.,* 2021). Large strandings of *Velella* as far north as British Columbia have been associated with El Niño (Schoener and Fluharty, 1985; Ware, 1995). At finer spatial scales (tens of meters), dense concentrations of *Velella* at sea are associated with convergent surface features (Shenker, 1988; Purcell *et al.,* 2012).

There is growing evidence of *Velella*’s ecological importance. For example, *Velella* have been reported as prey for turtles, fishes, janthinid snails and birds (Hubbs and Schultz, 1929; Harrison *et al.,* 1983; Hobson and Chess, 1988; Parker *et al.,* 2005). In addition, *Velella* may exert top-down influence by feeding on zooplankton and larval fishes (Bieri, 1961; Purcell *et al.,* 2012, 2015; Zeman *et al.,* 2018). Transport of *Velella* from ocean to land constitutes a substantial cross-ecosystem trophic subsidy (Kemp, 1986; Zuercher and Galloway, 2019).

**Fig. 1:**
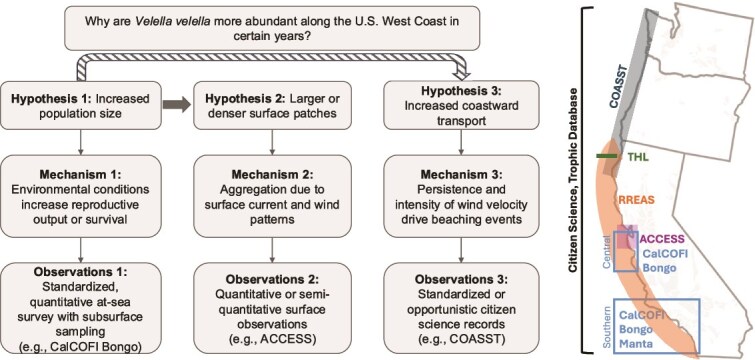
Hypothesized mechanisms related to changes in *Velella* abundance along the US West Coast. Hypotheses 2 and 3 may be linked to Hypothesis 1 as shown by thicker arrows. Not included is the effect of increased sampling effort over time. To test these hypotheses, specific types of data are required, which we compiled from various monitoring programs operating in different regions.

We present three non-exclusive mechanistic hypotheses linking population status and environmental conditions to increases of *Velella* along the US West Coast (Fig. 1): (i) increased population size; (ii) ocean currents and winds promoting aggregation and (iii) coastward transport from large-scale winds. Here, we combine available datasets to assess the testability of these hypotheses, noting the various datasets often span different spatial and temporal ranges that limit quantitative analyses (Supplementary Table S1). Our analysis highlights the recent abundance of *Velella* and explores factors that may contribute to these patterns. We provide recommendations for future monitoring and data integration to understand the utility of *Velella* as an ocean indicator.

## METHODS

### Data source overview

Although many surveys record *Velella* observations, no dedicated monitoring program exists to track their distribution and abundance along the US West Coast. To address this gap, we compiled data from the literature, oceanographic research surveys and citizen science programs (Fig. 1). These sources vary in the type of information provided, including abundance, presence/absence or other metrics (summarized in Supplementary Table S1).

### California Cooperative Oceanic Fisheries Investigations bongo

Annual mean densities (1951–2019, abundance m^−2^) of *Velella* at all phases and stages (1–40 mm) were obtained from nighttime bongo net tows conducted during spring (March–May) California Cooperative Oceanic Fisheries Investigations (CalCOFI) cruises (Lavaniegos and Ohman, 2007) and analyzed by the California Current Ecosystem Long-Term Ecological Research program (Ohman *et al.,* 2013). All tows were double oblique at a tow speed of ~0.75 m s^−1^ with mesh of either 550 or 505 μm with 333 μm mesh codend. Changes in sampling methods have been described by Ohman and Smith (1995), including a change from a 1-m diameter ring net beginning in 1951 to a paired bongo net with 0.71 m diameter openings in December 1977. The bongo net is 3 m in length, and is lowered at a 45° angle to 210 m, or 15 m from bottom at shallow stations, and remains at depth for 30 s. Subsamples from multiple stations within a survey were typically pooled for taxonomic analysis of organisms <25 mm (Lavaniegos and Ohman, 2007). Sampling was more frequent in Southern California (*n* = 64 years) relative to Central California (*n =* 44 years).

### CalCOFI Manta

A time series (1978–2010) quantifying proportional dominance of *Velella* in spring CalCOFI neuston samples was also available for Southern California (McClatchie, 2014). Surface plankton samples were collected using a manta net (mouth area of 0.133 m^2^, 505 μm mesh with a 333 μm mesh codend) that was towed at the surface for 15 min at a speed of ~0.75 m s^−1^ and catches were classified by the dominant plankton caught. A tow classified as “*Velella*” indicates that *Velella* was the dominant species, but a tow characterized by another species’ dominance or no dominance, does not preclude *Velella* from also being present. Time series were generated using proportions whereby the number of tows with *Velella* dominant in a year was divided by the total number of samples in that year.

### Applied California Current Ecosystem Studies

The Applied California Current Ecosystem Studies (ACCESS) surveys were conducted 3–4 times per year during April to October (excluding August) from 2010 to 2023 in north-central California. During standard seabird and marine mammal transects, *Velella* presence/absence and relative abundance was recorded in four categories: 0 = not present, 1 = present but rare, 2 = present and somewhat common and 3 = present and abundant.

### Trinidad Head Line

The Trinidad Head Line (THL) consists of five hydrographic stations (TH01–05) in northern California from the inner shelf to upper slope (Robertson and Bjorkstedt, 2020). The stations were sampled roughly monthly from 2006 to 2023. Initially, stations were sampled during darkness, but more recently in the late afternoon. *Velella* observations are not a part of THL protocols but are sometimes noted on datasheets.

### Global Biodiversity Information Facility

Opportunistic observations of *Velella* were downloaded from the Global Biodiversity Information Facility (GBIF) (GBIF, 2023). Data without location or date information were removed. This search contained 3360 observations from 18 sources in the study area; most of these records (98%) were research-grade observations (i.e. coordinates, community-confirmed identification) from “iNaturalist.”

### iNaturalist

A total of 4656 opportunistic *Velella* observations were downloaded from the iNaturalist citizen science application (www.inaturalist.org). Because research-grade observations from iNaturalist are incorporated into GBIF, this dataset was filtered to remove observations with the coordinates and dates matching observations from the GBIF dataset. Although these may be considered non-research-grade observations without community-confirmed identifications and coordinates, we chose to include them due to the conspicuous and widely known nature of *Velella* strandings and the fact that they are monotypic members of the genus, making misidentifications unlikely.

### Coastal Observation and Seabird Survey Team

Coastal Observation and Seabird Survey Team (COASST) is a citizen science program where expert-trained volunteers monitor sections of the coastline monthly for beachcast marine birds. Other data, including *Velella* presence, are collected opportunistically from 2000 to 2019 (see Jones *et al.,* 2021). Each site (*n* = 293) is fixed in space, marked permanently along the long axis of the beach.

### Rockfish Recruitment and Ecosystem Assessment Survey

The NOAA Rockfish Recruitment and Ecosystem Assessment Survey (RREAS) used visual surveys to describe the relative abundance of *Velella* in 2025. The survey had two legs (8–27 May and 31 May–17 June), each covering the California coast, and a sustained gale occurred between legs. During strip-transect seabird surveys, *Velella* observations were continuously recorded as an “intensity score” for relative abundance at 20 s intervals. The number of *Velella* within a ~3 m^2^ field of view looking over the bridge wing was estimated in four categories: 0 = none, 1 = 1–20, 2 = 21–100 and 3 ≥ 100 individuals.

Visual observations suggested that some seabirds may co-occur with *Velella*, particularly two species of phalaropes (red *Phalaropus fulicaria* and red-necked *Phalaropus lobatus*), which forage at-sea as spring migrants in California waters. We summed the total number of phalaropes across both species, as they are ecologically similar surface-feeding planktivorous foragers, and averaged phalarope counts and *Velella* intensities into 3 km bins. To test the hypothesis that phalaropes were positively related to *Velella*, we modeled positive phalarope counts (i.e. zeroes removed) as a smooth function of averaged *Velella* intensity using a generalized additive model (GAM, *mgcv* package in R). Phalarope counts were modeled with a negative binomial error distribution and log link to account for overdispersion in the count data.

### Trophic database

Predators of *Velella* were extracted from the California Current Trophic Database (Bizzarro *et al.,* 2023; Iglesias *et al.,* 2023) that spans 52 years of data from 10 surveys. The entire database was extracted from ERDDAP and then filtered by *Velella*’s id (117832) and grouped by predator name.

### Environmental analysis

Using data from the ship’s underway flow through system during the 2025 RREAS survey, we calculated the mean and standard deviation surface temperature, salinity and chlorophyll concentrations within the same 3 km bins as phalaropes and *Velella* to test whether *Velella* were associated with water properties (indicated by the mean) or frontal features (indicated by the standard deviation). Here, we consider surface frontal features to be gradients separating the boundaries of distinct water masses characterized by water properties, often due to strong convergence. We used linear regression to test for statistically significant relationships and acknowledge that 1 year of data limits statistical power. We also fit a GAM using the *mgcv* package in R to examine *Velella* responses to mean temperature and salinity, specifying a Tweedie error distribution to accommodate excess zeros and continuous positive values in the response variable.

Springtime *Velella* occurrence along the US West Coast is likely modulated by ocean–atmosphere conditions farther offshore in preceding months. To isolate environmental conditions associated with *Velella*, years or multiyear periods (“pulses”) when *Velella* were more abundant and widespread (based on literature and observations) were compared with other periods. To explore basin-wide atmospheric forcing, historical winds and mean sea-level pressure were obtained from the European Centre for Medium-Range Weather Forecasts ERA5 atmospheric reanalysis (Hersbach *et al*., 2020). For both, monthly anomalies were calculated relative to 1980–2024. Winds were calculated for each winter (January–March) within potential source regions to the southwest of California (20–40°N, 140–120°W) and in the vicinity of the North Pacific Current (40–50°N, 150–130°W), assuming 3-month travel at ~10–20 cm s^–1^ drift speed (Mackie, 1962). We also evaluated these patterns in relation to the Oceanic Niño Index (ONI) as a measure of El Niño-Southern Oscillation variability as well as the North Pacific High (NPH), which is a subtropical anticyclonic pressure system that drives upwelling along the coast (Schroeder *et al.,* 2009; Black *et al.,* 2011). For the latter, we determined the center position and area of NPH in January–February for each year (following Schroeder *et al*., 2013). We tested for significant differences in winds in both potential source regions as well as NPH area, latitude and longitude during pulse vs non-pulse years using a Welch’s two-sample *t-*test.

## RESULTS

Below, we first focused on spatial and temporal patterns from each sampling approach and program, and then we combined datasets to identify larger spatiotemporal patterns and investigate our hypotheses.

### Nets

**Fig. 2:**
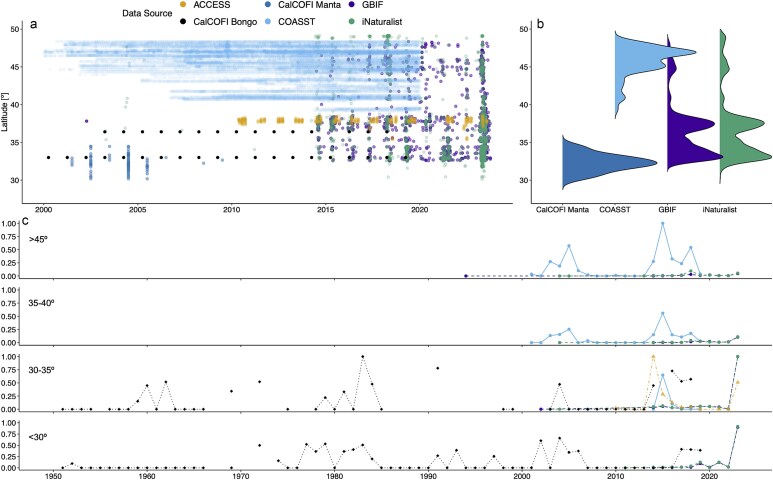
Temporal and spatial distribution of a subset of *Velella* datasets with longer time series. (a) The latitudinal coverage of datasets (colors) since 2000. (b) The latitudinal distribution of presences from the three data sources with spatially explicit data spanning at least 5° of latitude. For CalCOFI, manta net tows are shown. (c) Normalized time series in 5° latitudinal bins for comparisons of *Velella* occurrence and abundance between bins (rows) and data sources (colors). For COASST and ACCESS, time-series represent the ratio of presences to non-detections. For GBIF and iNaturalist, time-series represent the number of reported occurrences. For CalCOFI, time-series show log mean abundance values from bongo net tows pooled by year and region (i.e. south vs central), and thus are not shown in panels B.

CalCOFI bongo: Peaks in average annual spring densities were observed in Central California in 1983 (142 *Velella* m^−2^), 1991 (28.7 *Velella* m^−2^) and 2016 (19.6 *Velella* m^−2^; Fig. 2 and Supplementary Fig. S3A). Average annual densities were lower in Southern California than Central California, with a maximum average annual density in 2004 in Southern California (12 *Velella* m^−2^). Otherwise, average annual densities remained <10 *Velella* m^−2^ for both regions.

CalCOFI Manta: While the spring tows frequently captured no plankton (*n* = 1109 hauls) or no dominant type was established (230), when there was a dominant plankton type, *Velella* were the most frequently dominant (114); other dominant plankton types were salps (59), siphonophores (44), medusae (34) and ctenophores (9). The annual proportion of tows in which *Velella* dominated was relatively low and homogenous throughout the time series (mean of all years = 0.07 ± 0.13), except in 2002, 2004 and 2005 when *Velella* dominance spiked to >20% (max = 62% in 2004, Fig. 2 and Supplementary Fig. S3B).

### Citizen science

COASST: *Velella* were present in ~2% (465) of the 23 060 beach surveys (Fig. 2 and Supplementary Fig. S3C). There was interannual variability, with most sightings during 2003–2005 (*n* = 64) and 2014–2018 (*n* = 375). Most of the presences occurred in April (*n* = 170) and May (*n* = 120), while October, December and January had two records each. The number of *Velella* observations peaked in summer for 2014 (July: *n* = 17, August: *n* = 24), and there were few observations in June 2005 (*n* = 11) and 2015 (*n* = 16).

GBIF: A total of 3282 *Velella* presence records were retrieved from 1994 to 2023. Although individual occurrences were recorded in 1994 (*n* = 1) and 2002 (*n* = 2), the majority were found from 2014 onward with 67.5% of all records in 2023 (Fig. 2 and Supplementary Fig. S3D). The timing of *Velella* occurrences (Supplementary Fig. S1) varied with distinct spring pulses that were most evident in the northernmost (>45°N) and southernmost (<35°N) regions. The latter also had another peak in strandings in the late summer/early autumn, which was also reflected in the 35–40°N bin. The central regions displayed intra-annual distributions of occurrences ranging from the late spring to summer. Winter sightings, albeit rare (~4% of occurrences), were most common >45°N. The years with notable occurrences in July/August include 2014 and 2020–2023. The number of reports from September to December was generally low each year (*n* < 5).

iNaturalist: A total of 3354 observations distinct from those in GBIF were retrieved from 2003 to 2023. As with the GBIF data, the overwhelming majority (99.6%) of *Velella* were observed after 2014 (Fig. 2). Overall, the spatiotemporal patterns in iNaturalist observations tightly mirrored those in the GBIF data, with notable spring pulses, and summer observations largely occurring in 2014 and 2020–2023. Observations from September to December were rare, composing 1.4% of occurrences.

### Visual observations

**Fig. 3:**
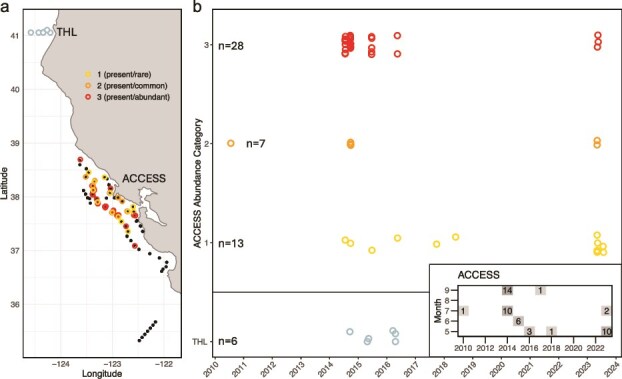
(a) Spatial and (b) temporal distribution of *Velella* observations from the THL and ACCESS surveys. The THL includes presence-only records (gray), while ACCESS includes three abundance categories. Black points are ACCESS stations where no *Velella* were observed. Inset (b) for ACCESS, the number of *Velella* observations by month and year.

ACCESS: *Velella* were present in 8% (*n* = 48) of the ACCESS observations, with 13, 7 and 28 records in abundance Categories 1–3, respectively (Fig. 3a). *Velella* were most frequently observed in 2014 and 2023 (73% of records) and to a lesser degree in 2015–2016 (18%, Fig. 3b). In years with multiple *Velella* observations, they were at least sometimes observed in higher abundances (e.g. Category 3, 2014–2016, 2023). *Velella* were observed in all survey months (May–July, September), with some years having more observations in May and others in September (Fig. 3b).

THL: There were six records of *Velella* presence on datasheets (Fig. 3). *Velella* were observed once in 2014 (September), twice in 2015 (April and May) and three times in 2016 (March and April, Fig. 3b), and at all hydrographic stations. Survey notes provided interesting anecdotes, such as the vessel went through “a cloud of *Velella*” in 2015, there were “millions of small, tiny *Velella* in mats” and *Velella* were “in the bongo net and not nearly as much phytoplankton” in 2016.

**Fig. 4:**
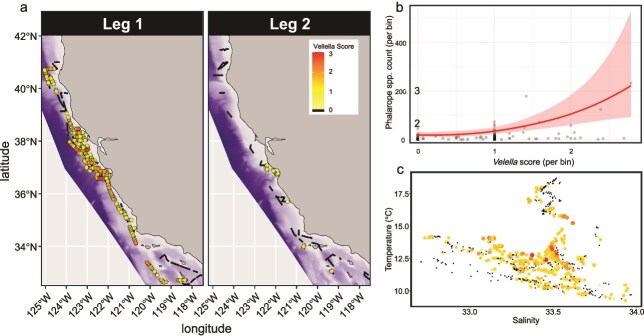
RREAS survey in 2025 with (a) *Velella* intensity scores shown during survey legs 1 and 2. Bottom depths range from 0 to 4000 m (lighter to darker shading). (b) Phalarope count where *Velella* were observed vs *Velella* intensity score. (c) Temperature-salinity diagram with the points colored by the *Velella* intensity score. Larger circles indicate higher intensities.

RREAS: During the first leg of the 2025 survey, *Velella* were observed throughout the entire survey but had nearly disappeared during the second leg, ~3 weeks later, perhaps related to a large gale between legs, with only a few locations with low intensity scores (Fig. 4a). During Leg 1, there was no clear spatial pattern in intensity scores as their distribution was patchy with both absences and high intensities in the north/south and onshore/offshore. The highest intensity patches (i.e. continuous *Velella* patches with scores ≥ 2) were infrequent and small, with intense patches 3 km long or larger representing only 3.4% of all *Velella* observations, and intense patches 6 km long or larger representing 2.1% of all *Velella* observations. Regions of lower *Velella* intensity (i.e. scores of 1–2) were slightly more extensive, with 8.4% of all *Velella* observations occurring within continuous low-intensity patches ≥50 km. Anecdotally, some patches were size-stratified with all individuals small or large in size.

### Predators and associates

Trophic database: The documented predators of *Velella* are blue rockfish (*Sebastes mystinus, n* = 51), Pacific hake (*Merluccius productus, n* = 1), Pacific jack mackerel (*Trachurrus symmetricus, n* = 39) and Pacific mackerel (*Scomber japonicus, n* = 3) (Supplementary Fig. S2 and Supplementary Table S2). Predators varied in spatial distribution with blue rockfish between 38 and 42°N and the other species between 46 and 47°N. Most records were grouped temporally: late 1970s to early 1980s, late 1980s and early 2000s, with the greatest number of observations documented from 2003 to 2005. Most observations occurred in May and June.

RREAS: In 2025, throughout the coast, Phalaropes were frequently observed in regions of high *Velella* abundance during the first survey leg. Phalarope sightings were mainly represented by red-necked Phalaropes (*n* = 1725) with red (*n* = 12) and unidentified Phalaropes (*n* = 8) more rarely recorded. Phalaropes were essentially absent during the second leg, likely explained by migratory timing as they moved north to breeding grounds. Focusing on Leg 1, Phalarope abundance was positively related to *Velella* intensity (Fig. 4b; edf = 1.0, *χ*^2^ = 10.6, *P* = 0.001, *R*^2^ = 6.4%).

### Timeline of *Velella* pulses

While *Velella* were observed in many years from ~1900 to 2025 along the US West Coast, years or multiyear periods (“pulses”) when *Velella* were more abundant and widespread included the early 1980s (1980–1984), the early 2000s (2002–2005), the mid-2010s (2014–2018) and 2023 (Figs 2 and 3b, Supplementary Fig. S3, and Supplementary Table S3). While *Velella* were observed in other years, they were either fewer (e.g. 2019–2022), temporally separated from these pulses (e.g. 1991, bongo and Trophic Database) and/or observational coverage was sparse (e.g. early 1950s, Bieri, 1959; Schoener and Fluharty, 1985).

### Environmental relationships

Spatial aggregation from RREAS: During Leg 1 in 2025, surface conditions varied with temperature ranging from 9.1 to 18.9°C, salinity from 32.2 to 34.0, and chlorophyll a from 0.5 to 2.6 μg L^−1^. We found *Velella* intensity scores were slightly higher when chlorophyll concentrations were more variable (linear regression, *t* = 1.7, *P* = 0.08, *n* = 757), mean salinity was higher (*t* = 1.8, *P* = 0.06, *n* = 761) and mean temperature was lower (*t* = −3.5, *P* = 0.0006, *n* = 761). Similarly, these *Velella* scores were significantly higher in higher mean salinity and lower to moderate temperature waters (Fig. 4c, GAM *P* < 0.05, deviance explained = 14.4%). Where *Velella* scores were highest was representative of cool, high salinity water characteristic of the spring/summer upwelling season (Fig. 4c).

**Fig. 5:**
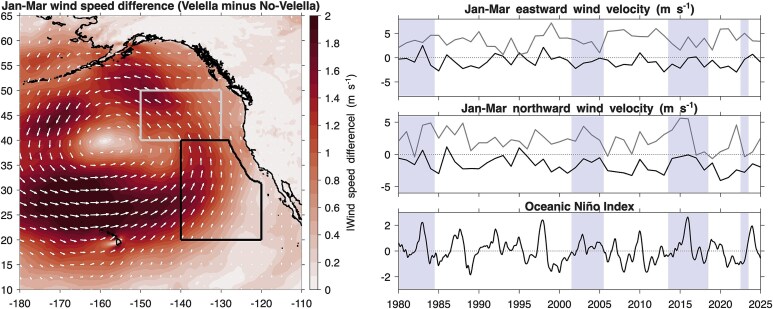
Northeast Pacific winds relative to years of elevated *Velella* presence along the US West Coast. (Left) Mean differences in January–March wind speed (color) and direction (arrows) between years with and without elevated *Velella.* Two possible *Velella* source region boxes are outlined. (Right) Time series of eastward and northward winds in each of the two offshore regions, as well as the ONI. Periods of elevated *Velella* presence (“pulses”) are shaded (1980–1984, 2002–2005, 2014–2018, 2023).

**Fig. 6:**
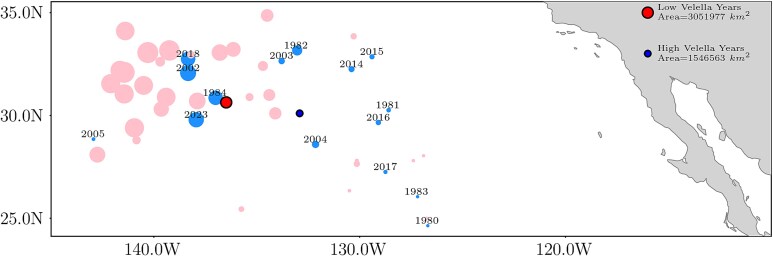
The center position of the NPH pressure system during the winter (January–February average). Marker size is proportional to the area of the NPH, with larger marker size denoting larger area. Years with high *Velella* presence (“pulses”) are labeled with the year. The climatological position and area for high and low *Velella* years is shown by the darker circles, see the legend for the value of the climatological area. During years when the NPH is smaller and closer to shore, upwelling along the coast is relaxed, sea surface temperatures are warmer, sea levels are higher and spring-time biological productivity is reduced (Schroeder *et al.,* 2013).

Wind patterns and pulse years: On average, pulses of *Velella* along the US West Coast coincided with cyclonic surface wind speed anomalies during winter, promoting transport toward the coast from the southwest (Fig. 5). However, offshore wind time series in presumed source regions of *Velella* did not always show a consistent correspondence with pulse years. In the region south and west of California (black outline in Fig. 5), shifts toward more northward and eastward winds were apparent during El Niño events (i.e. positive ONI), but El Niño does not appear to be a reliable driver of high *Velella* years along the coast. In the potential south source region, northward wind velocities were significantly less negative during pulse years (−1.07) than during non-pulse years (−1.87), indicating slightly weaker southward winds during pulses (*t-*test, *t* = 2.37, *P* = 0.023). In the potential north source region, eastward wind velocities were significantly lower during pulse years (3.17) compared to non-pulse years (4.04), indicating slightly weaker eastward winds during pulse years (*t*-test, *t* = −2.03, *P* = 0.049). The wind anomaly patterns (Fig. 5) were also associated with the strength and position of the NPH. During *Velella* pulse years, the winter NPH had a significantly smaller area (*t*-test, *t* = −2.79, *P* = 0.008), and the longitudinal center was closer to shore (*t*-test, *t* = 2.22, *P* = 0.035) (Fig. 6). Not all pulse years were associated with cyclonic wind anomalies (e.g. 1984 and 2002), with these years having a large NPH area.

## DISCUSSION

The ability to detect coastwide *Velella* presence relied on multiple monitoring efforts, open-access data and the collaboration of researchers. There has been an unprecedented number of at-sea observations and strandings since 2014, suggesting there have been changes to environmental conditions or in *Velella* vital rates, such as productivity or survival. The finding that *Velella* tend to occur in multiyear pulses raises the question of whether a single strong recruitment year may increase abundance in following years. Future work on aggregating and augmenting existing sampling programs could shed light on *Velella* life history, habitat preferences and ecosystem dynamics.

### Mechanisms of occurrence

The three hypothesized mechanisms (Fig. 1) likely collectively influence the detectability of *Velella*. High densities of gelatinous zooplankton along shorelines and convergent oceanographic features are commonly observed, but distinguishing physical aggregation from population increase is difficult (Graham *et al.,* 2001). Fronts associated with bathymetric shelf breaks or mesoscale eddies may concentrate organisms throughout the water column (Greer *et al.,* 2015; Schmid *et al.,* 2020) and could influence all life stages of *Velella*, but such signals are not always apparent (Luo *et al.,* 2014). In addition to wind forcing, surface slicks formed by internal waves may concentrate neuston at fine spatial scales and/or transport individuals shoreward (Shanks, 1983; Whitney *et al.,* 2021). Photographs of an at-sea patch of *Velella* in the northwest Mediterranean Sea enabled length measurements of colonies and the back-calculation of colony age based on assumed growth rates (Betti *et al.,* 2026). These authors suggested that larvae surfacing throughout a 3-month period were aggregated together in the same patch (Betti *et al.,* 2026). This observation supports the importance of subsurface sourcing paired with surface concentration mechanisms for high coastal densities of *Velella* to emerge. The consistent temporal alignment of increased subsurface medusae density and increased surface observations suggests increased population density within the California Current during pulse periods.

With an increased population size, larger or more numerous surface patches of *Velella* might be expected. We did not find significant relationships between *Velella* aggregation intensity and simple oceanographic frontal metrics from the single year of RREAS visual observations. This suggests spatial aggregation mechanisms are occurring but may require multiple years of data, more sophisticated frontal algorithms or a finer scale approach to test environmental relationships (e.g. Paduan and Washburn, 2013). However, the ACCESS sightings and THL anecdotal observations demonstrate that in years when *Velella* were more common, they were also found in higher densities. Further, given that *Velella* patches were anecdotally size-stratified suggests colonies remain in aggregations for prolonged periods, consistent with observations in the Mediterranean Sea (Betti *et al.,* 2026). Various relationships have been described to characterize associations between patch characteristics and total abundance of pelagic species. Acoustically derived biomass estimates of Antarctic krill and schooling fishes (herring, sprat, anchovy and sardine) share a common positive, linear relationship with the number of aggregations but not aggregation size (Brierley and Cox, 2015). By analogy, such a scaling relationship would imply that increased frequency of *Velella* patches likely indicates a larger regional population. Analysis of *in situ* optical observations of taxonomically diverse plankton demonstrated the relationship between aggregation count and mean population density can be parabolic, whereby the number of patches declines at particularly high densities, presumably as aggregations merge with one another (Robinson *et al.,* 2021). As such, the spatial extent of *Velella* patches could be an informative variable in addition to total number of patches and within-patch density.

Observations of *Velella* in fish diets and spatial associations with seabirds could also be indicative of patch dynamics. When predators encounter patchy dense prey, this can result in increased feeding rates (e.g. Benoit-Bird *et al.,* 2013; Gliwicz and Maszczyk, 2016). While our analysis focused on Phalaropes, it is possible that other seabirds are also co-located with *Velella* patches due to available prey resources concentrated in frontal zones (e.g. Ainley *et al.,* 2005; Scales *et al.,* 2014). More years of overlapping observations would be valuable to test this possible relationship. Documenting the myriad associations between *Velella* and vertebrate predators further supports the growing recognition that gelatinous organisms are important prey items for many taxa, both specialists and opportunistic generalists (Hays *et al.,* 2018).

Increased population size could result in more beaching events, or increased beaching events could be independent of population size and solely be related to coastward transport. Offshore wind anomalies during *Velella* pulses along the US West Coast tend to favor transport toward the coast from offshore and to the south, consistent with the hypothesized link between larger-scale atmospheric forcing and *Velella* transport to the eastern boundary of the North Pacific. Wind forcing was also hypothesized to be the dominant driver of *Velella* distributions more locally along the Portuguese coast where relaxation of upwelling was implicated in coastward transport (Pires *et al.,* 2018). The North Pacific Current, another potential source of *Velella* to the US West Coast, results in surface ocean transport directed eastward toward the continental margin, potentially supplying organisms to multiple regions along the coast. However, the mean wintertime winds in that region would favor transport toward the Canadian/Alaskan coast (e.g. Fiedler and Mantua, 2017), and wind anomalies in the region during *Velella* pulses are away from the coast (Fig. 5).

The position and area of the NPH seems to be a more reliable indicator of coastal *Velella* observations. Because our time series of *Velella* are incomplete and *Velella* may not have been detected in scientific surveys, high *Velella* years may have gone undetected. Consequently, years characterized by an eastward NPH position and reduced spatial area were classified as low-*Velella* years, perhaps weakening the statistical relationship between the two (Fig. 6). For all of the hypotheses, the disparate nature of the data compiled here leads us to be cautious about overinterpretation.

Although our study focuses on the drivers of spring detections of *Velella*, since this was when the majority of observations occurred, occasional summer and fall detections also occurred (Bieri, 1977; Jones *et al.,* 2021). Notably, widespread summer-early Fall observations occurred in 2014 across programs (THL, ACCESS, COASST, GBIF and iNaturalist). In 2013, a multiyear marine heatwave (2014–2016) began to develop, with a large, persistent warm-water anomaly in the northeast Pacific in late 2013 that spread into the California Current in 2014 (Di Lorenzo and Mantua, 2016). The development of this heatwave was associated with an unusually persistent high-pressure system along the US West Coast (the “Ridiculously Resilient Ridge”; Swain *et al*., 2017), present for much of 2013 and 2014 (Supplementary Fig. S4), which weakened the climatological westerlies in the region (Bond *et al.,* 2015). This event caused major ecosystem disturbances with increased species richness and unusual species distributions (“tropicalization”) (Leising *et al.,* 2015). In addition, citizen science and ACCESS data suggested additional summer anomalies in the early 2020s and particularly in 2023, although we did not have scientific survey data for all datasets during this time. Unlike in 2014, productivity was initially high in 2023 and warming developed later in association with El Niño, resulting in more moderate ecosystem impacts. Given that *Velella* were more regularly on the beaches and reported in the media in recent years, it is difficult to determine how anomalous the early 2020s were from citizen science observations alone.

### Integration of diverse datasets

Disentangling the hypothesized drivers of the occurrence of *Velella* remains challenging. Spatial and temporal survey coverage was patchy, often with limited overlap between programs. The complicated and poorly understood life history of *Velella* requires study on the distribution across life stages, but data are scarce, particularly during the medusa phase. Despite the useful insights from citizen science data, the opportunistic and unstructured nature of these efforts (e.g. GBIF, Dickinson *et al.,* 2010) can also contribute to spatial biases (Rapacciuolo *et al.,* 2021). We found little difference between GBIF and the non-research grade iNaturalist observations, suggesting that observations without community-confirmed identifications may be equally useful for conspicuous taxa, such as *Velella*. Citizen science data were primarily coastal and near human population centers, while more systematically collected datasets (e.g. CalCOFI) extend further offshore but do not reach the hypothesized population center in the central Pacific. Additional sources of bias arise from different gear types, sampling approaches and pooling samples in space/time (e.g. CalCOFI). Moreover, inconsistent measurement units (e.g. presence-only, presence–absence, abundance) make synthesizing the data a challenge. Integrated species distribution models that incorporate multiple data types are one promising approach to quantifying various sources of error and potentially alleviating these biases for improved distribution predictions and understanding of environmental drivers (Miller *et al.,* 2019; Mäkinen *et al.,* 2024).

## CONCLUSIONS AND FUTURE DIRECTIONS

Recommendations for future work: A priority is to resolve the source regions and transport pathways that deliver *Velella* to the coast. For example, Lagrangian particle-tracking experiments, forced by realistic surface ocean currents and winds, could reconstruct likely trajectories and source regions by tracing their trajectories backward from the stranding locations. If *Velella* are transported as colonies (rather than at depth as medusae), incorporating sail dynamics (Mackie, 1962; Bourg *et al.,* 2024) into particle tracking experiments could improve mechanistic understanding of *Velella* transport. Once source regions are better constrained, targeted analyses can evaluate which environmental conditions favor high *Velella* abundance. This approach could be applied to other transient species (e.g. pyrosomes, Hetherington *et al.,* 2026) and has already been used to understand red crab range expansions (Cimino *et al.,* 2021).

At least four sampling programs have archived *Velella* samples that could be analyzed to produce, in some cases, a multidecadal time series of abundance, biomass, length frequency, life history and other characteristics (Supplementary Information). Zooplankton community information could also be obtained from these samples to understand whether *Velella* co-occur with other species or during times of higher/lower species richness. These additional datasets may also allow for further investigation into match–mismatch dynamics concerning periods when *Velella* were observed both offshore and on the beach vs only observed offshore.

Recommendations for sampling programs: At-sea survey programs should systematically record unusual species detections, such as those of *Velella* (Snell Taylor *et al.,* 2018). Integrating these observations into structured surveys, such as seabird and marine mammal sightings (e.g. RREAS and ACCESS) would provide more quantitative data. Simple presence–absence can be recorded by untrained observers, but abundance categories are more useful. With advances in artificial intelligence and image processing, automated counts could also provide valuable *Velella* data. As climate change and variability increases, species once considered unusual may become useful indicators of ecosystem changes—making it important to document these observations now.

Understanding long-term change: Despite observational inconsistency, coherent multiyear pulses of *Velella* along the US West Coast over the last five decades provide a valuable perspective for understanding long-term change more broadly. In the future, shifts in the phenology and biogeography of *Velella* may prove to be more readily detectable than changes in population growth or abundance (cf., Epinoux *et al.,* 2026). Looking forward, temperature and seasonal wind shifts (Jones *et al.,* 2021) are the key candidate drivers of variability in the abundance, distribution and phenology of *Velella* along the US West Coast. While the mystery of the by-the-wind sailor is still playing out, there are many avenues to advance understanding of *Velella* as widespread sighting reports in April–May 2026 continue to inspire further investigation.

## Supplementary Material

Cimino_et_al_Velella_Supplemental_Information_-_revision2_fbag044

## Data Availability

Data from bongo net tows is available from the Cooperative Zooplankton Dataspace of the CCE-LTER program (http://oceaninformatics.ucsd.edu/zoodb/). The California Current Trophic Database is available at https://oceanview.pfeg.noaa.gov/erddap/tabledap/SWFSC-CCTD.html.
