# Supplementary material for: Diverse sampling programs highlight pulses of Velella velella along the US West Coast
Source: J Plankton Res. 2026 Jun 13;48(4):fbag044. doi: 10.1093/plankt/fbag044 (PMC13264391; doi:10.1093/plankt/fbag044)
Supplement: Cimino_et_al_Velella_Supplemental_Information_-_revision2_fbag044 [file cimino_et_al_velella_supplemental_information_-_revision2_fbag044.docx]

**SUPPLEMENTARY INFORMATION**

***Additional Information on Data Sources***

During our search for *Velella* records, we also found that many programs do not record any information on *Velella*, such as Reef Check (https://www.reefcheck.org/), Beachcombers (https://mlml.sjsu.edu/beachcombers/), and NOAA Coastal pelagic species surveys (epipelagic trawls). While *Velella* may be observed during these monitoring efforts or appear incidentally in nets, no formal records are available. Further, *Velella* have not yet been detected in seawater environmental DNA (eDNA) samples taken regularly from Monterey Bay, California and across the California Current ecosystem (Kathleen Pitz, *pers. comm.*). We also queried various media sources (e.g., X, news articles) for reports of *Velella*, but found citizen science programs captured these observations. We downloaded high-resolution satellite imagery when large surface patches were observed but we are lacking reflectance measurements of *Velella.* We hope to acquire this information in the future as ship-based spectral radiometers can collect this measurement. Synthetic Aperture Radar (SAR) may be another useful tool for detection that we did not investigate here.

In addition, several surveys have collected *Velella*, but the associated data were not readily available. In Oregon, the Cooperative Research Program funded quarterly sampling along the Newport Hydrographic (NH) line from 2015–2021 generated 186 neuston samples, most of which are unprocessed (Toby Auth, *pers. comm.*). Similarly, the Pre-recruit survey project collected at least 406 unprocessed neuston samples along multiple transects off the Oregon and Washington coast during the spring of 2014–2019 (Curtis Roegner, *pers. comm.*). The Pelagic Invertebrate Collection (PIC) at Scripps Institution of Oceanography also has specimen jars from 1978 to present that may contain *Velella*. Finally, the Trinidad Head Line has specimens in jars. Although it was beyond the scope of this project to process these archived samples, they may contain valuable information on *Velella* for future work.

***Summary of Sightings from Literature Review***

During the 1926 El Niño event, *Velella* were reported in large numbers both at sea and washed ashore from Cape Mendocino, northern California to Hecate Strait, British Columbia (Hubbs & Schultz, 1929; Ware, 1995 and references within). These reports coincided with increased observations of ocean sunfish from Oregon to British Columbia, and stomach content analysis of one individual from Washington indicated it fed heavily on *Velella* (Hubbs and Schultz 1929). Although *Velella* beachings have been documented in Washington in several other years (e.g., 1927, 1934, 1956, 1957, 1959), these events were generally of low abundance. In contrast, notably large strandings occurred in 1965, 1970, 1981, and 1983-1984, the latter associated with an El Niño event (Schoener & Fluharty, 1985 and references therein). Kemp (1986) reported scattered *Velella* individuals along central Oregon beaches in 1982, 1983 and 1985, in contrast to mass strandings recorded in 1981 and 1984, focusing detailed observations on three strandings in May 1984.

Mackie (1962) collected specimens at the surface and onshore in Bodega Bay, northern California in June 1960 to conduct experiments on sailing speed and direction. Bieri (1977) summarized surface observations in the eastern North Pacific and strandings along the North American Pacific coast from 1889-1975 to calculate growth rates and describe the species’ life history.

*Velella* were present in diet samples of blue rockfish *Sebastes mystinus* from April to September 1978-1981 in coastal waters off Mendocino, northern California (Hobson & Chess, 1988). Analysis of media reports and citizen science data from the JellyWatch Project suggested more prevalent strandings of *Velella* along the North American Pacific coast in 2014 compared to the preceding four years (Purcell *et al.*, 2015). In May and June 2015, *Velella* were opportunistically sampled from a bloom offshore of Oregon and southern Washington (Zeman *et al.*, 2018).

**REFERENCES**

Bieri, R. (1977) The ecological significance of seasonal occurrence and growth rate of *Velella* (Hydrozoa). *Publ. Seto Mar. Biol. Lab.*, **24**, 63–76.

Hobson, E. S. and Chess, J. R. (1988) Trophic relations of the blue rockfish, *Sebastes mystinus*, in a coastal upwelling system. *Fish. Bull.*, **86**, 715–743.

Hubbs, C. L. and Schultz, L. P. (1929) The northward occurrence of southern forms along the Pacific Coast in 1926. Calif. Fish Game, 15, 234–240.

Kemp, P. F. (1986) Deposition of organic matter on a high-energy sand beach by a mass stranding of the cnidarian Velella velella (L.). Estuar. Coast. Shelf Sci., 23, 575–579. https://doi.org/10.1016/0272-7714(86)90010-0.

Mackie, G. O. (1962) Factors affecting the distribution of *Velella* (Chondrophora). *Int. Rev. Gesamten Hydrobiol. Hydrogr.*, **47**, 27–36.<https://doi.org/10.1002/iroh.19620470104>.

Purcell, J. E., Milisenda, G., Rizzo, A., Carrion, S. A., Zampardi, S., Airoldi, S., Zagami, G., Guglielmo, L., Boero, F., Doyle, T. K. and Piraino, S. (2015) Digestion and predation rates of zooplankton by the pleustonic hydrozoan *Velella velella* and widespread blooms in 2013 and 2014. *J. Plankton Res.*, **37**, 1056–1067.<https://doi.org/10.1093/plankt/fbv071>.

Schoener, A., and Fluharty, D.L. (1985) Biological Anomalies off Washington in 1982-83 and other Major Niño Periods. In: El Niño North: Niño Effects in the Eastern Subarctic Pacific Ocean. W.S. Wooster and D.L. Fluharty (eds.). Seattle: Wash. Sea Grant, Univ. Wash., pp. 211-225.

Schroeder, I. D., Black, B. A., Sydeman, W. J., Bograd, S. J., Hazen, E. L., Santora, J. A. and Wells, B. K. (2013) The North Pacific High and wintertime pre-conditioning of California Current productivity. *Geophys. Res. Lett.*, **40**, 541–546. https://doi.org/10.1002/grl.50100.

Ware, D. M. (1995) A century and a half of change in the climate of the NE Pacific. Fish. Oceanogr., 4, 267–277. https://doi.org/10.1111/j.1365-2419.1995.tb00073.x

Zeman, S. M., Corrales-Ugalde, M., Brodeur, R. D. and Sutherland, K. R. (2018) Trophic ecology of the neustonic cnidarian *Velella velella* in the northern California Current during an extensive bloom year: insights from gut contents and stable isotope analysis. *Mar. Biol.*, **165**, 150. https://doi.org/10.1007/s00227-018-3404-1.

**SUPPLEMENTARY TABLES**

**Table S1.** Overview of data sources used in this study, including survey and data type, *Velella* life stage, spatial and temporal extent of each dataset, and a summary of the number of observations obtained.

| **Data Source** | **Survey Type** | **Data Type** | **Life stage** | **Spatial Extent** | **Temporal Extent** | **Number of Observations** |
| --- | --- | --- | --- | --- | --- | --- |
| **ACCESS** | At-sea | presence-absence & relative abundance | Adult | 37.57-38.13 ºN; upper continental slope to coastal waters <15 m from shore | 2010-2023 | 525 absence, 48 relative abundance |
| **COASTT** | Beach | presence-absence | Adult | ~40.35-48.5 ºN | 2000-2019 | 458 presences and 22602 presumed absences |
| **CalCOFI Manta** | At-sea | dominance | Adult | ~ 29.8-34.5 ºN | 1978-2010 | 114 Velella dominant, 1485 not dominant |
| **CalCOFI Bongo** | At-sea | Density [log(abundance m-2 +1)] | Hydranth - Adult [~1-40 mm] | Regionally pooled in Southern and Central California regions (see Fig 1) | 1951-2019 | 119 |
| **GBIF** | Beach | presence-only | Adult | CCE | 1994-2023 | 3282 |
| **THL** | At-sea | presence only; not consistently recorded | Adult | 41.05 ºN; 5-37 km from shore; 35-780 m depth | 2006 -2023 | 6 |
| **RREAS** | At-sea | presence-absence & relative abundance | Adult | ~32-41ºN | 2025 | Leg 1: 439 bins where present, 369 bins where absent; Leg 2: 31 bins where present, 319 bins where absent |
| **Trophic Database** | At-sea | presence-only | Adult | ~ 38-47 ºN | 1977 - 2005 (12 unique years) | 94 |
| **iNaturalist** | Beach | presence-only | Adult | ~ 30-49 ºN | 2003-2023 (16 unique years) | 3354 |

**Table S2.** Summary of *Velella* predators identified in the NOAA SWFSC California Current Trophic Database as of 05 September 2025 pooled by year, survey/organization, and predator common name. The month(s) is also listed. The database contains diet information from 105,694 individual predators among 143 taxa, including marine mammals, fishes, and squids.

| **Survey/Organization** | **Predator** | **Year** | **Number of Observations** | **Month(s)** |
| --- | --- | --- | --- | --- |
| NMFS-SWFSC-FED | Blue Rockfish | 1977 | 2 | 6 |
| NMFS-SWFSC-FED | Blue Rockfish | 1978 | 4 | 5,6 |
| NMFS-SWFSC-FED | Blue Rockfish | 1979 | 2 | 4,8 |
| NMFS-SWFSC-FED | Blue Rockfish | 1981 | 12 | 5,6 |
| NMFS-SWFSC-FED | Blue Rockfish | 1986 | 3 | 6 |
| NMFS-SWFSC-FED | Blue Rockfish | 1987 | 9 | 5 |
| NMFS-SWFSC-FED | Blue Rockfish | 1988 | 15 | 4,6 |
| NMFS-SWFSC-FED | Blue Rockfish | 1991 | 4 | 5 |
| OSU-CIMRS/NMFS-NWFSC-EOEP | Pacific Hake | 2004 | 1 | 6 |
| OSU-CIMRS/NMFS-NWFSC-EOEP | Pacific Jack Mackerel | 1999 | 2 | 7 |
| OSU-CIMRS/NMFS-NWFSC-EOEP | Pacific Jack Mackerel | 2003 | 37 | 6 |
| OSU-CIMRS/NMFS-NWFSC-EOEP | Pacific Mackerel | 2005 | 3 | 6 |

**Table S3**. Summary of multi-year periods (pulses) when *Velella* were more abundant and widespread from our review of multiple data sources.

| **Pulse years** | **Sources** |
| --- | --- |
| 1980-1984 | CalCOFI bongo, Trophic Database, Schoener & Fluharty, 1985, Kemp, 1986 |
| 2002-2005 | CalCOFI bongo, CalCOFI manta, COASST, Trophic Database, GBIF |
| 2014-2018 | CalCOFI bongo, COASST, GBIF, ACCESS, THL, iNaturalist, Purcell *et al.,* 2015, Zeman *et al.,* 2018 |
| 2023-? | GBIF, ACCESS, iNaturalist, RREAS (Santora pers. obs.) |

**SUPPLEMENTARY FIGURES**


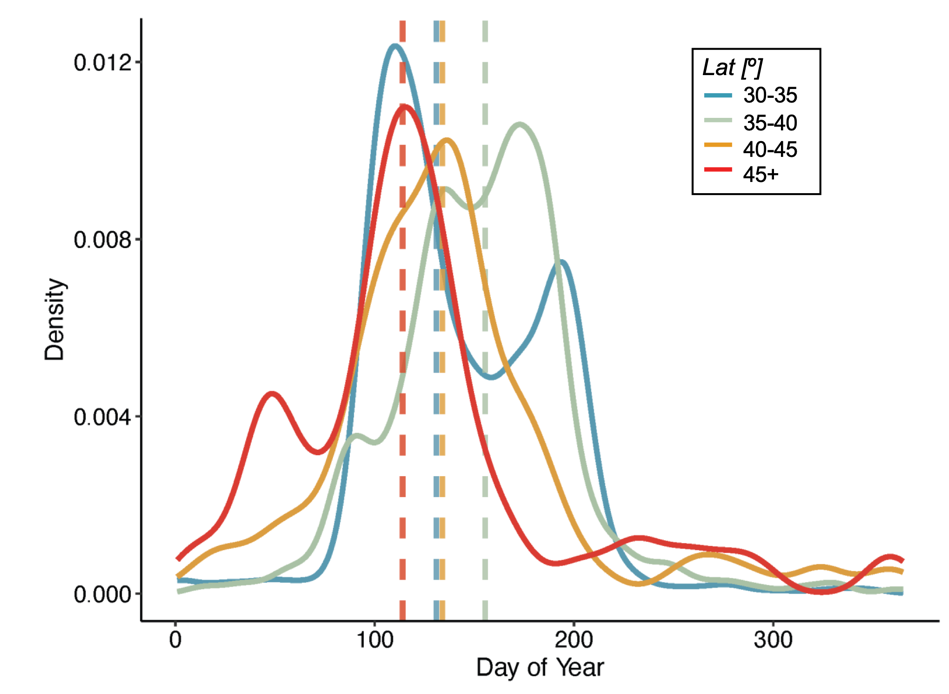


**Figure S1**. Intra-annual timing of *Velella* strandings by latitude (5º bins) in GBIF. Solid lines and vertical dashed lines show kernel densities of day-of-year and median day-of-year of occurrence, respectively.

**Figure S2.** Spatial distribution and relative density of *Velella* predators in the California Current reported in the NOAA SWFSC California Current Trophic Database as of 05 September 2025. The data are summarized in greater detail in Table S2.

**
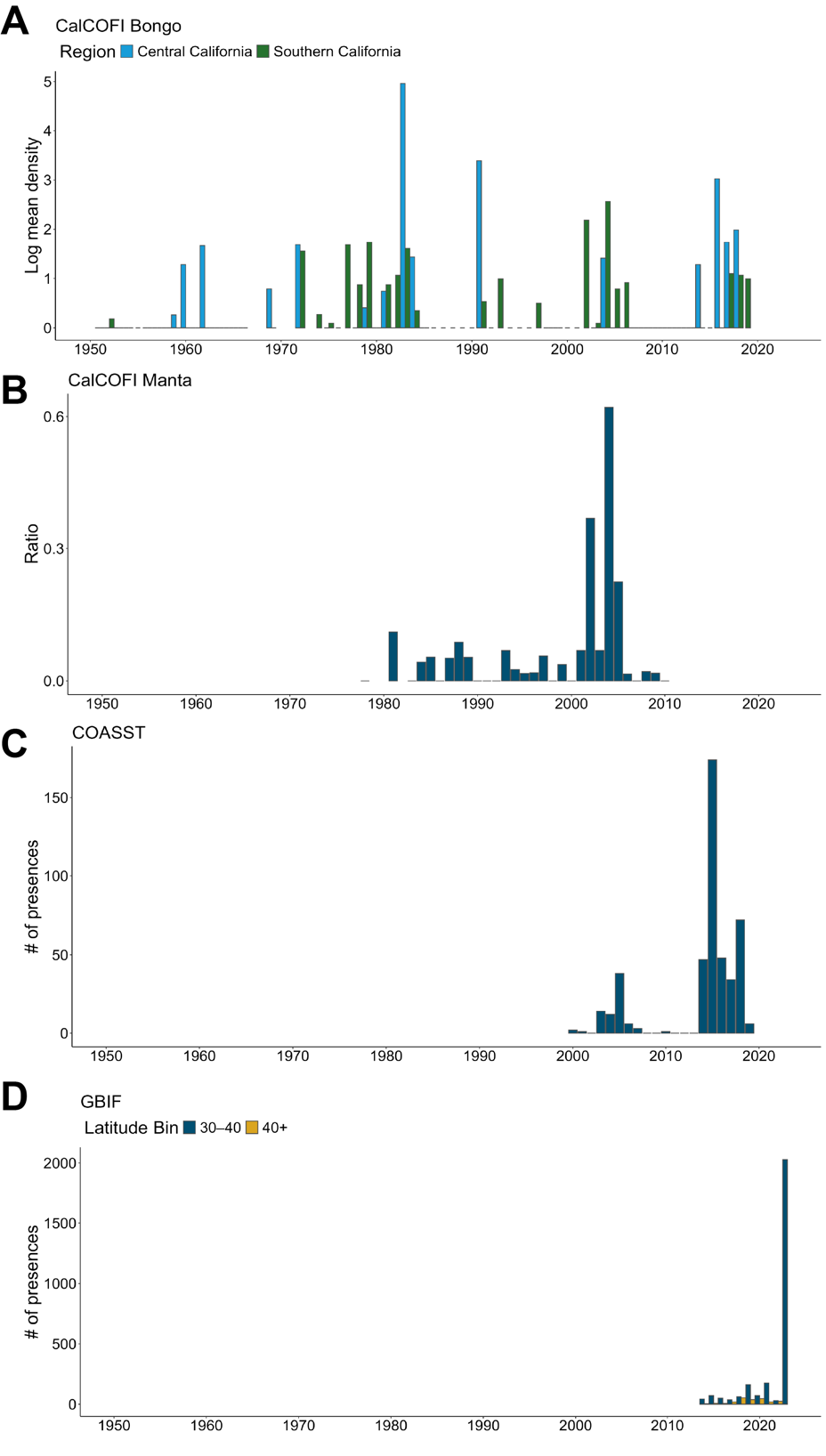
**

**Fig. S3.** Time series of *Velella* datasets. **A)** Mean annual densities (log density + 1) of *Velella* collected during the spring CalCOFI bongo net surveys separated by Central and Southern California. **B)** The annual proportion of spring CalCOFI surface manta tows in which *Velella* were the dominant gelatinous zooplankton species in southern California. **C)** The annual number of presences from the monthly COASST beach surveys from northern California to Washington. **D)** Number of *Velella* beach strandings from GBIF citizen science reports along the U.S. West Coast.


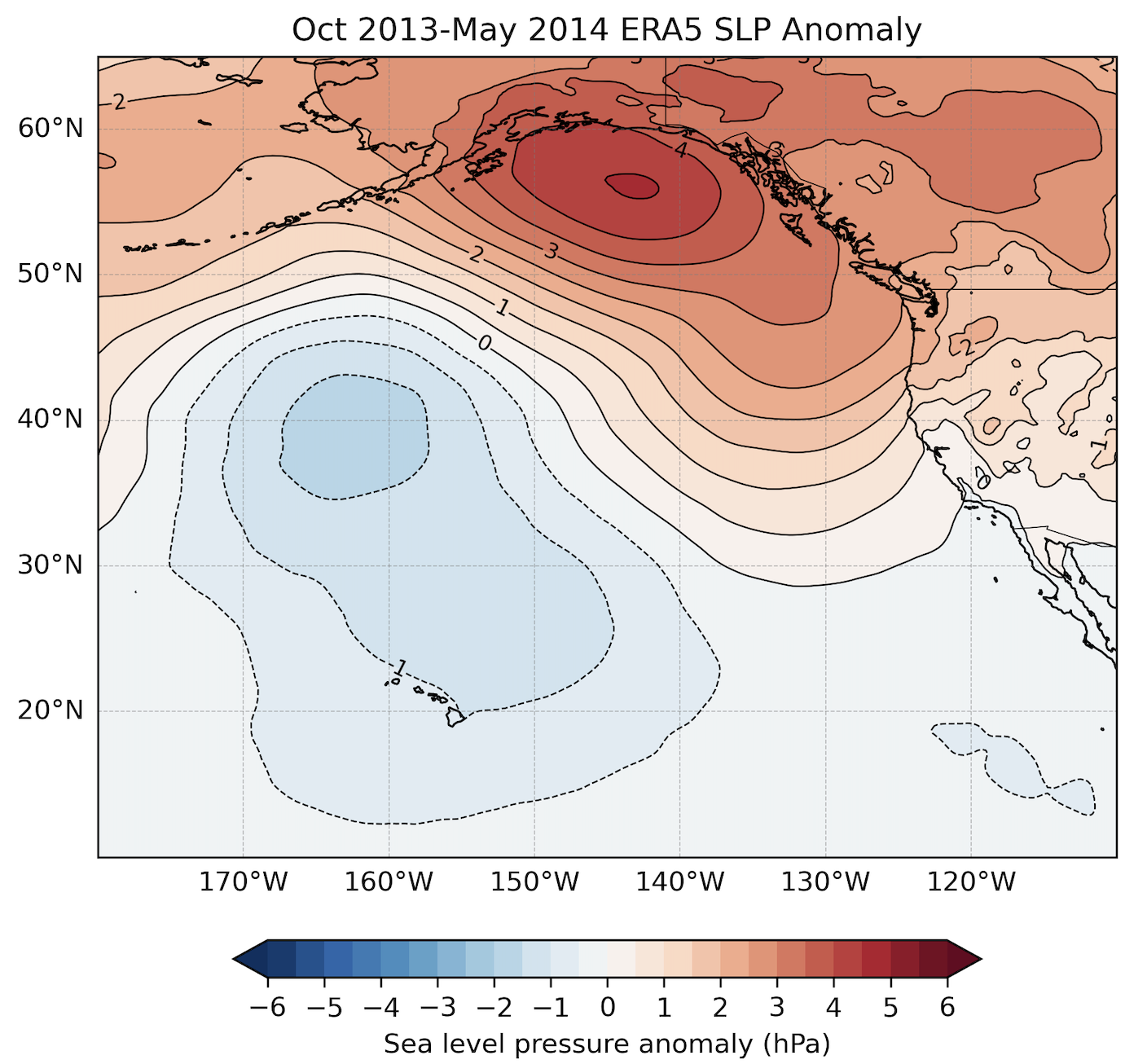


**Fig. S4.** Sea level pressure anomaly with respect to the long-term mean (1980-2024) across the eastern Pacific during October 2013-May 2014.
